# Structure and Stability Insights into Tumour Suppressor p53 Evolutionary Related Proteins

**DOI:** 10.1371/journal.pone.0076014

**Published:** 2013-10-04

**Authors:** Bruno Pagano, Abdullah Jama, Pierre Martinez, Ester Akanho, Tam T. T. Bui, Alex F. Drake, Franca Fraternali, Penka V. Nikolova

**Affiliations:** 1 King’s College London, School of Biomedical Sciences, Department of Biochemistry & Randall Division of Cell and Molecular Biophysics, New Hunt’s House, London, United Kingdom; 2 Department of Pharmacy, University of Naples “Federico II”, Napoli, Italy; 3 Institute for Pharmaceutical Science, London, United Kingdom; University of Saarland Medical School, Germany

## Abstract

The p53 family of genes and their protein products, namely, p53, p63 and p73, have over one billion years of evolutionary history. Advances in computational biology and genomics are enabling studies of the complexities of the molecular evolution of p53 protein family to decipher the underpinnings of key biological conditions spanning from cancer through to various metabolic and developmental disorders and facilitate the design of personalised medicines. However, a complete understanding of the inherent nature of the thermodynamic and structural stability of the p53 protein family is still lacking. This is due, to a degree, to the lack of comprehensive structural information for a large number of homologous proteins and to an incomplete knowledge of the intrinsic factors responsible for their stability and how these might influence function. Here we investigate the thermal stability, secondary structure and folding properties of the DNA-binding domains (DBDs) of a range of proteins from the p53 family using biophysical methods. While the N- and the C-terminal domains of the p53 family show sequence diversity and are normally targets for post-translational modifications and alternative splicing, the central DBD is highly conserved. Together with data obtained from Molecular Dynamics simulations in solution and with structure based homology modelling, our results provide further insights into the molecular properties of evolutionary related p53 proteins. We identify some marked structural differences within the p53 family, which could account for the divergence in biological functions as well as the subtleties manifested in the oligomerization properties of this family.

## Introduction

The p53 protein family plays a key role in many different biological functions spanning different aspects of health and disease [1,2]. Perhaps the most exciting development in the ever growing complexity of p53 functions is the recognition that its ability to act as a “guardian of the genome” and hence to prevent cancer has evolved relatively late, its early functions being the protection of germ-line integrity and monitoring development [1,3]. Furthermore, transcriptional profiling data from *C. elegans* suggests that the DNA damage dependent apoptosis is an ancient function of the p53 family [4]. This interplay of intrinsic p53 functions contributes to the increased complexity of the network of genes implicated in its regulation. The ability of the p53 protein family to elicit cell cycle arrest and apoptosis is clearly one of the most dynamic of functions as it has a direct link to tumour suppression and cancer biology [5,6]. A proper understanding of the evolution of the p53 gene necessitates the analysis of the homologous p63 and p73 genes and their products as it is now widely accepted that the entire family is at the centre of a complex network charged with responding to signals in diverse cellular functions. A genome-wide study showed that p63 and p73 regulate a range of unique target genes involved in multitude of biological functions including DNA repair [7]. Specifically, p63 and p73 transcriptionally up-regulate BRCA2, Rad 51 and mre11 and hence provide a new mechanism for the action of the p63 and p73 in tumour suppression. Based on sequence alignment, invertebrate p53 shows greater similarity to p63 than to mammalian p53 or p73. Amongst these, the *Drosophila melanogaster* (p53_fly) gene codes for a single p53-like protein, which stands as an ancestor of the mammalian p53 family of proteins, making it of particular importance for evolutionary studies. Furthermore, the p53_fly gene is crucial to preserve genomic stability by regulating cell death, which makes it, in terms of function, more similar to human p53 than to p63 or p73. It has been reported that this gene incorporates multiple aspects and diversities of the functions associated with the p53 family of proteins [8]. Furthermore the p53 protein from the Placozoans (*Trichoplax adhaerens*) is significantly closer to human p53 than the corresponding sequences of p53-like proteins from the fruit fly *D. melanogaster* and the nematode *C. elegans* [9]. It is speculated that since both of these proteins have been shown to retain p53-like functions (i.e. DNA damage-induced apoptosis) it is therefore likely that the newly discovered *Trichoplax* gene will result in a protein with p53-like biological function(s). It should be noted that the amino acid sequence identity between the human p53 DBD and that of the *Trichoplax* is 38% [9].

A powerful approach to better understand the multitude of functions displayed by the p53 protein family is through evolutionary analyses. New techniques have been applied in a search to find the fundamental determinants of protein evolution [10,11]. Advances in systems biology together with functional and structural genomics are enabling the characterization of global cellular networks [12]. A novel approach integrating time dimensionality in p53 protein-protein interaction networks predicted four distinct binding sites in the p53 DNA-binding domain [13]. This study reported at least 12 different proteins as potential ligands for these four binding sites within the p53 DBD. Interestingly some of these interactors can bind at the same time while others cannot. It was proposed that Ku70, Cdk7 and RAP1A bind to p53 through the same site and therefore cannot bind to p53 at the same time.

Could there be further insights that we can learn from the p53 evolution that might contribute to the elucidation of the rapidly emerging functions and the mechanisms of actions of these proteins? The deleterious effect of disease-associated mutations are said to be susceptible to selective pressures, the same pressures that have most likely contributed to sequence conservation during evolution [11,14]. The amino acid substitutions that are disease-associated are suggested to be those that are different in nature from those normally acquired during evolution of closely related species [15]. If so, it might be plausible to exploit such knowledge in designing strategies for therapeutic applications.

Interestingly, species like the subterranean blind mole rat *Spalax* has evolved unique p53 function during 40 million years of hypoxic life [16]. Specifically, *Spalax* p53 was reported to favour growth arrest but not apoptotic target genes [16,17]. Sequence alignment showed some key differences, namely Arginine residues at position 174 and 209 in human p53 correspond to Lysine residues in *Spalax*. These amino acid substitutions are key for the observed differences in function between *Spalax* and human p53. The report showed that “humanizing” *Spalax* p53 by site-directed mutagenesis at position R174 resulted in 3-fold increased activation of apaf 1 promoter, whereas changing lysine to arginine at position 209 did not produce any effect. However, when both amino acids were changed to that of the human p53 the effect was synergetic, leading to 5-fold increase relative to the wild-type *Spalax* p53. These observations are particularly significant given that Arginine at codon 174 substitution to Lysine is reported to be observed in a wide range of different tumours including that of breast, colon, lung and liver cancers. Similarly, Arg 209 is mutated in a range of tumours including substitutions of Arg to Lys, including that of skin, colon, and oesophageal cancers.

Here, we investigate and build upon the information related to the structure of the p53 family of proteins. It is known that the *Homo sapiens* p53 protein [18-22] consists of a central DNA-Binding domain (DBD), a tetramerization domain (TD), a trans-activation domain (TA) as well as a C-terminal regulatory domain. The highest amino acid sequence identity across different species is observed in the central core DBD. In terms of stability, it is well known that the p53 DBD displays unusually low thermodynamic stability compared to its homologues p63 and p73 [23-25]. Computational studies have been performed to understand the molecular basis of p53 thermodynamic instability [26-28] and identified ways to enhance the p53 stability. We aimed to further understand the intrinsic role of the structure and stability in p53 protein evolution by comparing a number of selected species. We investigated some members of the p53 protein family with special focus on protein structure, biochemical and biophysical properties of the DNA binding domains of the selected proteins and their conformational dynamics. For this purpose, we provide detailed analysis of the p53 ancestry of seven different protein family members by comparing the secondary structures, folding, thermal stabilities (using the apparent melting temperatures, T_m_) and complement these studies with comprehensive computational investigations including sequence-based homology modelling as well as molecular dynamics simulations. The proteins considered here are the following: *Homo sapiens* p53 (p53_human), *Mus musculus* p53 (p53_mouse), *Gallus domesticus* p53 (p53_chicken), *Drosophila melanogaster* p53 (p53_fly), *Caenorhabditis elegans* p53 (p53_worm), *Homo sapiens* p63 and p73. We discuss our findings in the context of the newly published structures of p53, p63 and p73 and possible implications for functions [21,22,29,30].

## Materials and Methods

### Protein cloning, expression and purification

The pRSET (A) modified plasmid (Invitrogen) was used for high-level expression of all proteins used in *E. coli C41* (DE3) cells (Avidis). Plasmid constructs encoding the DNA binding domains of *Homo sapiens* p53 (residues 94-312), *Homo sapiens* p63 (residues114-326), *Homo sapiens* p73 (residues 104-333), *Mus musculus* p53 (residues 91-308), *Gallus domesticus* p53 (residues 87-278), *Caenorhabditis elegans* p53 (residues 220-420) and *Drosophila melanogaster* p53 (residues 78-277) were used for protein expression using methods as described in Patel et al., 2008 [24]. The boundaries of the proteins were selected based on the multiple sequence alignment of all the proteins using p53_human as a reference. Briefly, the overnight cells grown in 2xTY media supplemented with Ampicillin and induced with 1 mM IPTG were harvested at 4 °C. The proteins were extracted using BugBuster Protein Extraction Reagent (Novagen) and Benzonase nuclease following the manufacturer instructions and supplementing the buffer with 1 protease inhibitor tablet (Roche) per 50 ml extraction reagent. The buffer used was 50 mM Tris HCl, 5 mM DTT with pH 7.2. Proteins were incubated for 10 minutes at room temperature, centrifuged at 13,000 rpm for 25 min at 4 °C. The supernatant was filtered and used for protein purification on AKTA PRIME connected to PRIMEVIEW software. We used a three-step purification method involving ion exchange SP chromatography, followed by affinity high performance Heparin chromatography and finally HiLoad 26/60 Superdex 75 gel filtration chromatography. The purified proteins were flash frozen in liquid nitrogen and stored at -80 °C for further use.

### Circular Dichroism to probe the secondary structure and folding as well as the thermal denaturation of the proteins

Circular Dichroism (CD) was used to assess the secondary structures of the proteins of interest. The scans of the purified proteins were acquired on a Chirascan Spectropolarimeter (Applied Photophysics). Samples were prepared in phosphate buffer (10 mM Na P, pH 7.2, 150 mM NaCl, 4 mM DTE) and filtered using sterile 0.2 µm filter (Whatman). The protein concentrations used were 0.2 mg/ml. The Far-UV CD spectra were acquired using the following parameters: wavelength ranges 260-190 nm, spectral bandwidth-1nm, step size-0.5 nm, time per point 3.0 seconds. The CD spectra of each protein was recorded at 20 °C, cooled to 4 °C, heated to ~ 90 °C and re-cooled to 20 °C. The instrument was equipped with a Melcor Thermoelectric Peltier unit set to change the temperature from 4-98 °C at a rate of 2 °C/min with a 2 °C step size and 0.2 °C tolerance. A 10 s time-per-point was used. The temperature was measured directly with a thermocouple probe in the protein solution. All measurements were performed in 0.5 mm cell path length. All spectra were corrected for the buffer baseline. The protein secondary structure estimation was calculated using the Principle Regression method.

### Structure-based homology modelling

**Figure 1:**
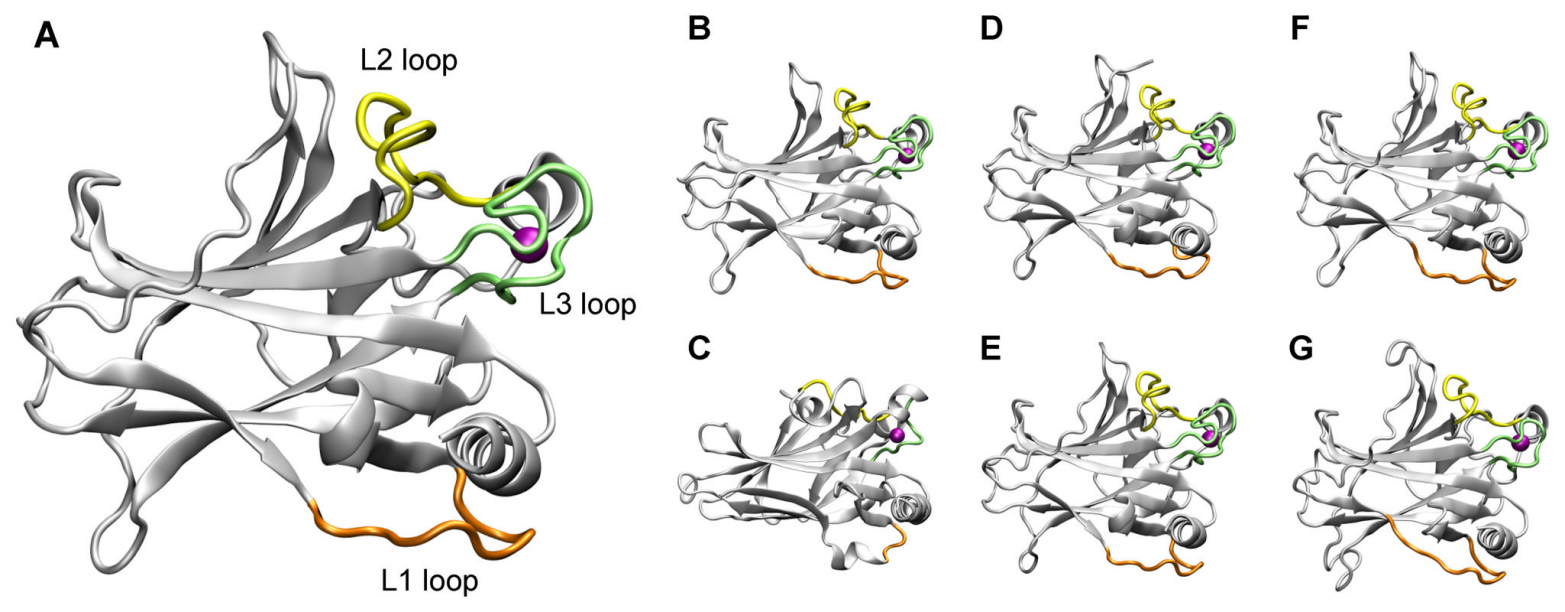
Ribbon representation of the 3D structures used for the bioinformatics analysis and MD simulations. Ribbon representation of the crystal structures of p53_human (A), p53_mouse (B) and p53_worm (C) and of the model structures of p63_human (D), p73_human (E), p53_chicken (F) and p53_fly (G). L1, L2 and L3 loops are highlighted in orange, yellow and lime, respectively. The zinc ion is represented as a purple sphere.

The 3D structures used for the analysis and simulations are respectively: *Homo sapiens* p53 (pdb code 2OCJ), *Mus musculus* (pdb code 1HU8) and *C. elegans* (pdb code 1T4W) [20,31,32] (Figure 1A-C). The model structures of *Homo sapiens* p63 and p73, *D. melanogaster* p53 and *Gallus domesticus* p53 (Figure 1D-G) were obtained by homology modeling from the crystal structure of *Homo sapiens* p53, with which they share about 60% sequence identity. This allows for a reliable model, considering that a sequence identity threshold for obtaining a satisfactory comparative homology model is 30% [33].

During the course of this work, 3D structures have become available for p63 and p73. We have therefore inspected the structures for p63 (pdb code 3US0) [34] and p73 (pdb codes 3VD2, 2XWC, 4A63, 4G82) [30,35] and compared with our models presented here. We have calculated the RMSD between our initial model and these structures and we observed a good fitting with values ranging from 1.2 Å to 6.0 Å (for the p63 with bound DNA, structure 3US0), see Figure S1. It has to be considered that some of these structures are solved with bound DNA and therefore expectedly different in the binding loops.

Sequence alignments were generated using the T-Coffee program [36]. 3D models were generated using the MODELLER package [37]. 200 models were generated for each protein and the ones with the best DOPE function score were selected for further studies. To refine the models, energy minimizations were performed with the GROMACS package [38] using the GROMOS96 force field [39]. GROMACS package and self-written programs have been used for the analysis of the data. Images were produced with the Visual Molecular Dynamics (VMD) 1.8.7 [40] and Pymol [41] programs.

### Bioinformatics analysis

#### Structural alignment

The MAMMOTH server [42] was used to produce the structural alignment of the structures and generated models.

#### Hydrogen bond wrapping

Backbone hydrogen bonds (BHB) were extracted from the structure files using the DSSP software [43] with the standard cut-off on the bond energy (-0.5 kcal/mol). The "wrapping" was computed as described by Fernández and Scheraga [44], counting the number of hydrophobic CH groups within the desolvation area of the hydrogen bond. A BHB desolvation area is defined by two spheres of 6.5 Å centred on the two residues C-alpha atoms. Vulnerable hydrogen bonds (Vbonds) were defined as the bonds in the tail of the wrapping distribution, in agreement with Fernández and Scheraga definitions [44]. The background wrapping distribution was obtained by running the analysis on the transient and obligates complexes as listed by Mintseris and collaborators [45-47]. A maximum of 16 CH groups in the desolvation area was determined as threshold for the Vbond definition.

#### Residue average exposure and interface propensity

We used the set of non-redundant interfaces from Keskin et al. [48] to compute the average residue exposure and interface propensity. Solvent-accessible surface areas (SASA) were computed using the POPS software [49,50] and interface residues were determined as the residues that show a 10% reduced SASA compared to the fully exposed state calculated with POPSCOMP [51]. The probability of an amino acid involved in an interface is given by p = (number of occurrences of amino acids located at an interface / number of instances of the amino acids).

### Molecular Dynamics simulations

MD simulations on the DNA-binding domains were performed with the GROMACS package [38] using the 53A6 parameter set of the GROMOS96 force field [39]. Molecules were neutralized with Cl^-^ ions (placed following electrostatic potential values) and solvated in boxes containing about 12200-14600 SPC (simple point charge) water molecules [52]. Initially, water molecules and ions were relaxed by energy minimization and allowed to equilibrate for 200 ps of MD at 300 K with the solute molecules restrained at their initial geometry with a force constant of 3000 kJ mol^−1^ nm^−2^. The bonds were constrained by the LINCS algorithm [53]. Finally, the equilibrated systems were subjected to unrestrained MD simulation for 30 ns. Simulations were carried out with periodic boundary conditions at a constant temperature of 300 K. The Berendsen and v-rescale algorithms were applied for pressure and temperature coupling respectively [54,55]. The PME (Particle Mesh Ewald) method was used for the calculation of electrostatic contribution to non-bonded interactions (grid spacing 0.12 nm) [56]. MD trajectories were analyzed by using GROMACS analysis tools. The Dynamite server [57] was used to produce principal component analysis (PCA) of the MD trajectories.

Secondary structure assignment from atomic coordinates of proteins was obtained with the program STRIDE [58]. The Pymol molecular graphics software was used for the execution of APBS to calculate electrostatic potentials and the visualization of the resulting electrostatic surfaces [41,59]. The ConSurf server was used to obtain the evolutionary conservation pattern of residues of the p53_human DBD structure [60]. Images were produced with VMD (version 1.8.7) and MOLMOL (version 2K.2) molecular visualization programs [40,61].

## Results

The choice of p53 evolutionary related proteins in this work has been made to include the DBDs of different species, representing proteins from vertebrates p53_human, p53_mouse, p53_chicken as well as invertebrate species like p53_fly and p53_worm orthologs. For completeness, human p63 and p73 proteins have also been included. All protein constructs were expressed in *E. coli* as described before [24].

### Assessment of the folding and the apparent melting temperatures as a measure of the thermal stability of the p53 family of proteins by CD spectroscopy

**Figure 2:**
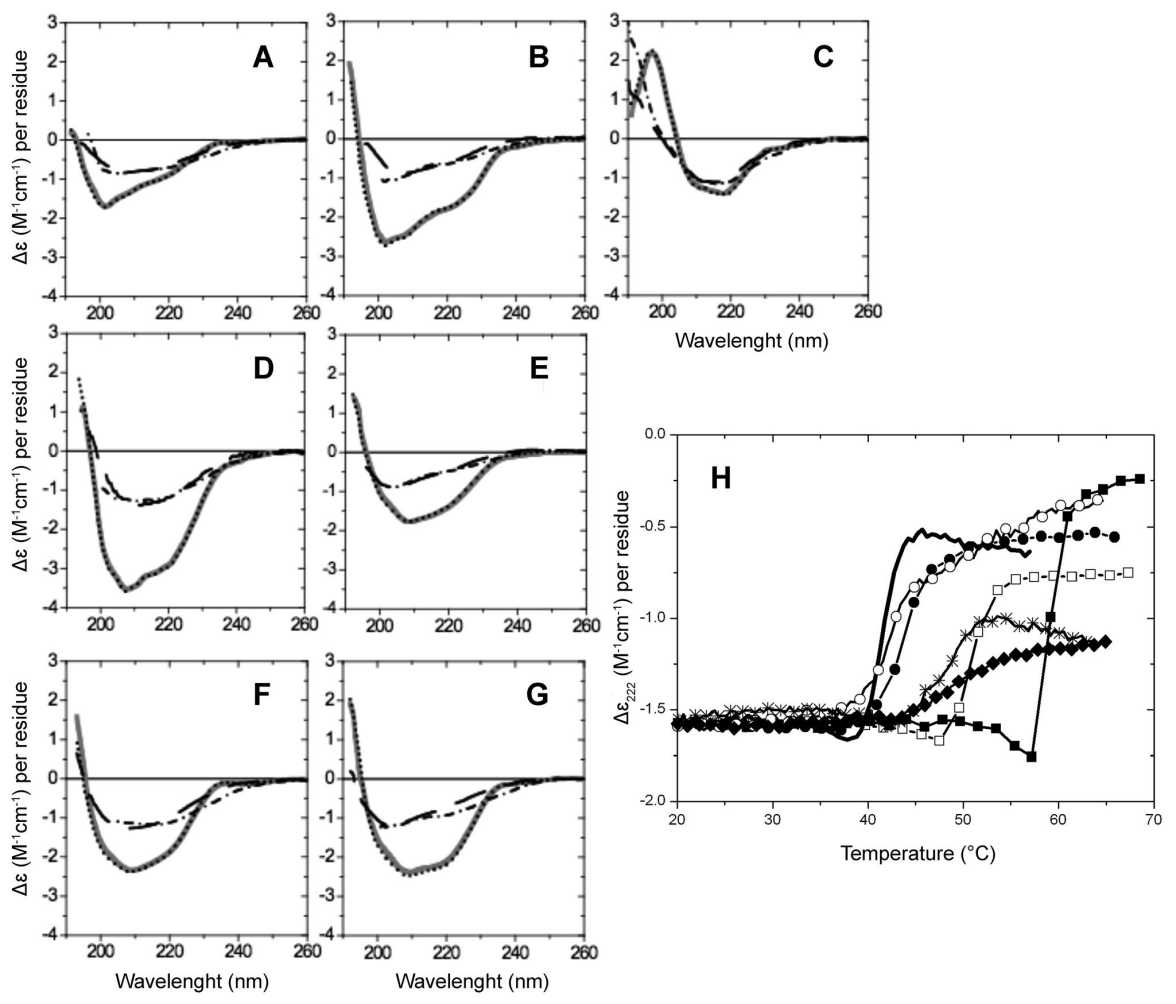
Far-UV CD spectra of p53 family of proteins (A-G) and CD thermal melting curves (H). CD spectra of (A) p53_human (res. 94-312), (B) p53_mouse (res. 91-308), (C) p53_worm (res. 220-420), (D) p53_fly (res. 78-277), (E) p53_chicken (res. 87-278), (F) p63_human (res. 114-326), (G) p73_human (res. 104-333). Recorded at (-) 20°C, (····) 4°C, (-·-·-) 90°C, and at (----) 20°C after heating. (H) CD thermal melting curves of (-) p53_human, (-●-) p53_mouse, (-∨-) p53_worm, (-○-) p53_fly, (-♦-) p53_chicken, (-■-) p63_human, (-□-) p73_human.

To assess protein folding and secondary structure content, far UV-CD spectra were recorded at different temperatures (20, 4, 90 and cooling back to 20 °C) (Figure 2A-G). The calculated secondary structure contents are listed in Table 1. The far UV-CD spectra of the p53 protein family DBDs at 20 °C and 4 °C (after cooling) show the typical signature of folded proteins with significant β-sheet content and a lesser amount of α-helix (Figure 2A-G), as observed for the X-ray structures of p53 DBDs.

**Table 1:** Secondary structure content estimation of the p53 protein family derived from the CD spectra and the MD structures (averages over 30 ns).

**Protein**	**Temperature**	**α-helix %**	**β-sheet %**	**Other**	**MD (ST) α-helix %**	**MD (ST) β-sheet %**
p53_human	20 °C	**3.1**	**38.8**	58.1	**2.6 (8.2)**	**38.1 (36.1)**
	4 °C	3.3	38.9	57.8		
	92.5 °C	6.4	33.2	60.4		
	20 °C after 92.5 °C	2.6	38.1	59.3		
p63_human	20 °C	**13.3**	**36.0**	50.7	**6.1 (7.1)**	**36.2 (34.2)**
	4 °C	13.5	34.6	51.9		
	92 °C	10.1	31.7	58.2		
	20 °C after 92 °C	6.3	37.7	56.0		
p73_human	20 °C	16.2	27.5	56.3	5.2 (6.8)	41.1 (35.4)
	4 °C	16.7	27.4	55.9		
	96 °C	8.1	32.9	59.0		
	20 °C after 96 °C	3.4	38.2	58.4		
p53_mouse	20 °C	13.5	30.4	56.1	2.7 (6.4)	39.2 (36.0)
	4 °C	13.7	28.5	57.8		
	94 °C	5.4	33.9	60.7		
	20 °C after 94 °C	1.6	39.7	58.7		
p53_fly	20 °C	23.7	25.6	50.7	5.1 (8.1)	43.1 (34.5)
	4 °C	23.4	24.6	52.0		
	89.6 °C	10.5	31.4	58.1		
	20 °C after 89.6 °C	7.6	36.2	56.2		
p53_chicken	20 °C	9.8	36.1	54.1	4.1 (7.7)	42.0 (34.4)
	4 °C	9.1	36.8	54.1		
	92 °C	4.1	34.4	61.5		
	20 °C after 92 °C	0	41.0	59.0		
p53_worm	20 °C	**9.6**	**39.0**	51.4	**12.8 (17.9)**	**40.3 (39.3)**
	4 °C	9.5	39.7	50.8		
	87.4 °C	5.0	42.1	52.9		
	20 °C after 87.4 °C	5.4	40.5	54.1		

Values for the initial structures (ST) are given in parenthesis. In bold the values that strongly correlate with the experimentally measured ones.

To test for the reversibility of folding, CD spectra were measured during heating to ~90 °C and after cooling back to 20 °C. All proteins unfolded irreversibly, the CD spectra at 20 °C before and after heating to 90 °C were different. The α-helix and β-sheet contents changed during the heat/cool cycle as indicated in Figure 2A-G and Table 1. Notably, the percentage of “other” (unfolded) forms increased as the result of heating.

The apparent melting temperatures (T_m_) are reported in Figure 2H and Table 2. The T_m_ values vary significantly and are in increasing order (°C): p53_human -41.5, p53_mouse -44.0, p53_fly -47.3, p53_worm -47.8, p53_chicken -49.0, p73 -49.5 and p63 -59.0, respectively. The data imply that the highest apparent melting temperatures are that of p63 and the lowest that of p53 human. Within the studied p53 species, the p53_chicken exhibited the highest melting temperatures.

**Table 2:** Apparent melting temperature.

**Protein**	**T_m_ °C**
p53_human	41.5
p63_human	59.0
p73_human	49.5
p53_mouse	44.0
p53_fly	47.3
p53_chicken	49.0
p53_worm	47.8

### Molecular Dynamics simulations and structure-based homology modelling

To investigate the conformational flexibility and stability of the p53 family of proteins at molecular level we subjected the above proteins to detailed Molecular Dynamics simulations. We built structure-based homology models for those proteins whose structures were unknown at the time, to be able to perform simulations and to compare the structural stability of all the p53 homologs selected for this study. The aim was to dissect in detail the intrinsic factors that contribute to the protein stability of the various homologous proteins and extract conclusions on molecular features that contribute to the observed differences.

### Structure-based comparison of the p53 protein family

#### Oligomerization properties of the DNA-binding domain

**Figure 3:**
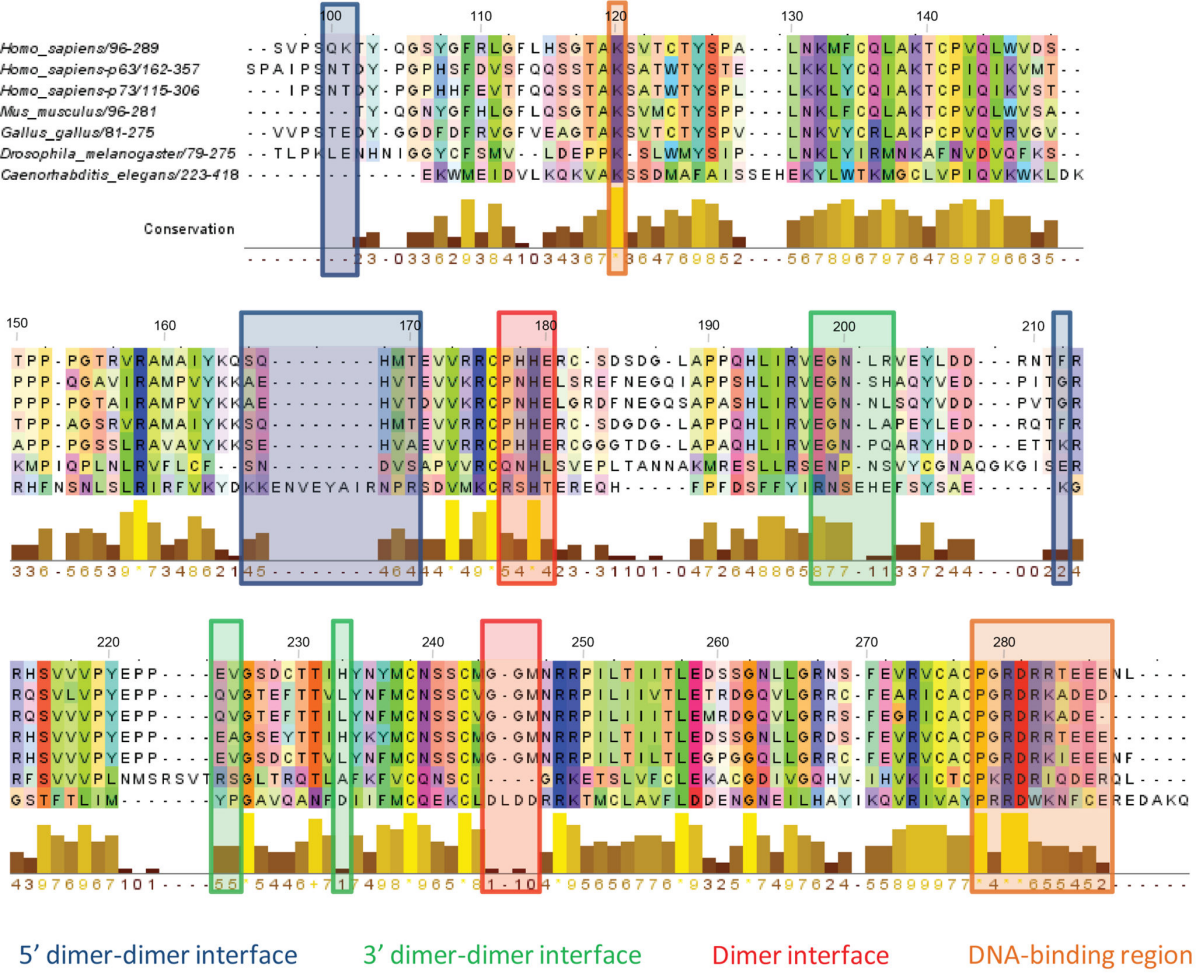
Multiple structure alignment of the p53 family. The alignment shows the conservation of the functional residues involved in zinc-binding and DNA-binding. The reported dimer and dimer-dimer interfaces are poorly conserved in *D. meloganaster* and *C. elegans*. An insertion in the zone reported to include the 5’ dimer-dimer interface residues of the isolated DBD is evident between residues 82 and 89. The lysine residue from loop 1 and terminal helix involved in DNA-binding are highlighted in orange.

The 7 orthologous structures were aligned using the MAMMOTH server [42]. Previously reported dimer interfaces (5' and 3') and dimer of dimers interfaces of the isolated DBDs [21] were mapped onto the structural alignment and highlighted in Figure 3 (shaded rectangles in blue, green and red). This alignment sheds light on different aspects of conservation in the orthologs. Functional residues involved in zinc-ion or DNA-binding are perfectly conserved in all 7 species. In the assigned dimer interface, the mouse, chicken as well as human p53, p63 and p73 paralogs present almost the same sequence. The only difference being a mutation of a histidine to an asparagine in the alignment position 178 for both p63 and p73. Specifically in this region, the p53_fly and p53_worm sequences are very divergent apart from the histidine residue involved in zinc binding. In the stretch close to the C-terminus, the sequence divergence is more apparent with the p53_worm sequence including one residue insertion and the p53_fly showing four residues deletion. For these two species, the 3’ and 5’ dimer of dimers interfaces also display high variability, especially for the 5’ dimer of dimers, where an 8-residue insertion is present in the worm sequence (area highlighted in blue in Figure 3).

#### Stability of the S1 structure in *C. elegans* and *Homo sapiens* p53

**Figure 4:**
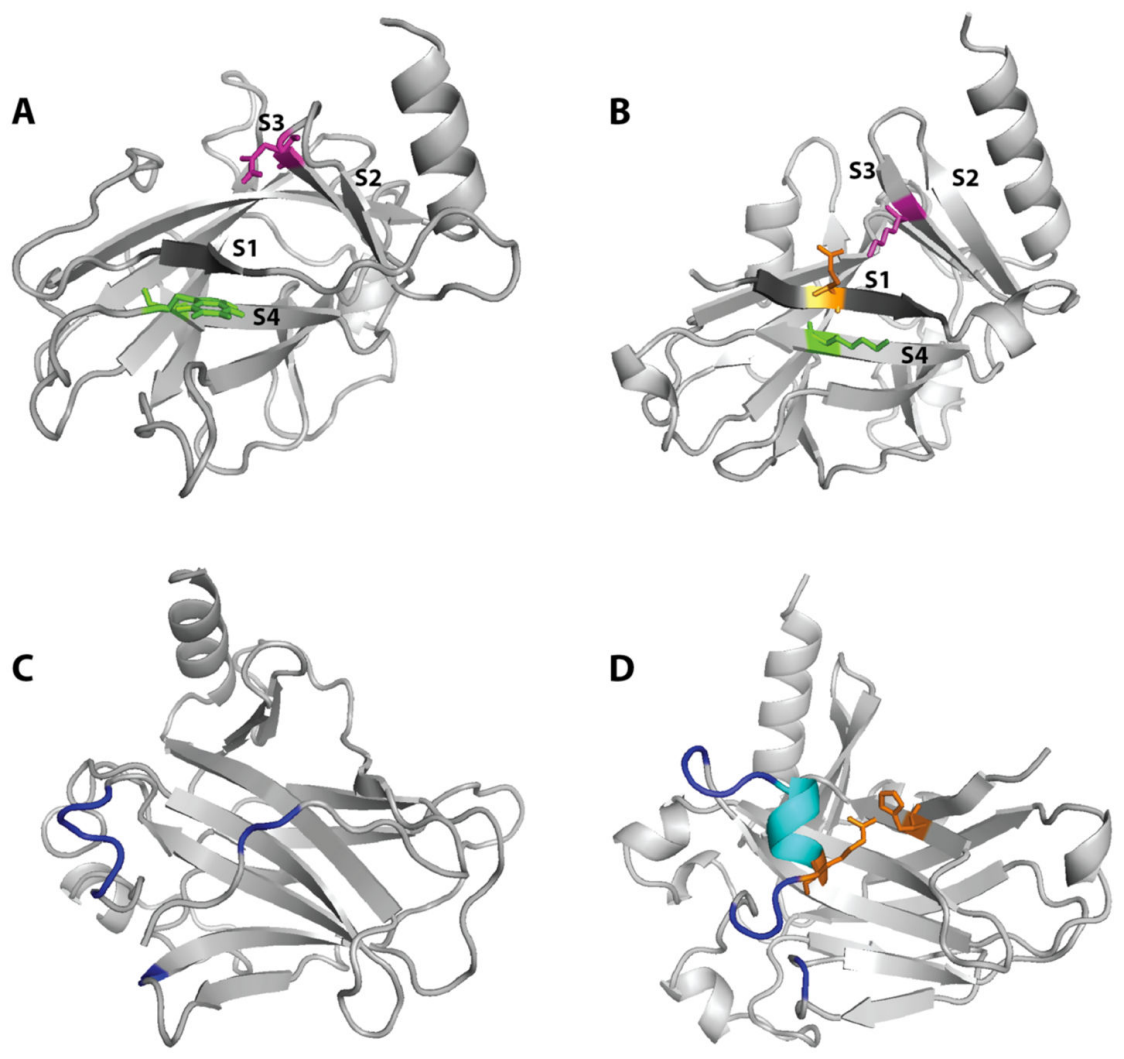
Structural divergences between human and worm p53 in the starting configurations for the Molecular Dynamics. (A) p53_human. Trp146 is highlighted in green and Asn131 in magenta, S1 is shown in dark grey. (B) p53_worm. Lys266 (aligned to *H. sapiens* Trp146) is highlighted in green and Lys251 (aligned to *H. sapiens* Asn131) in magenta, S1 is shown in dark grey. Glu227 (in orange) forms a salt bridge with Lys251, thus stabilizing the S1 and S3 secondary structures and is highlighted in orange. (C) p53_human. Residues involved in the 5’ dimer-dimer interface are highlighted in dark blue. (D) p53_worm. Residues involved in the 5’ dimer-dimer interface are highlighted in dark blue. Residues corresponding to the observed insertion in p53_worm are shown in cyan, and a helical structure is present, shielding the β-barrel core from the solvent. The proximity of residues Arg298 and His391 (in orange) may confer stability to this local conformation.

The β-sheet between strand 1 (S1) and strand 4 (S4) is elongated in the p53_worm structure due to the presence of two additional hydrogen bonds. In addition, the presence of a lysine (Lys266) instead of a tryptophan (Trp146 in p53_human) contributes to an increased stability of the DBD β-barrel core, due to the stronger propensity for β-sheet formation of Lys vs Trp (Figure 4).

In the p53_human X-ray structure (PDB code 2OCJ) the hydrophobic side chain of Trp146 is oriented towards the solvent, with a total of 86 Å^2^ of Solvent Accessible Surface Area (SASA) [49,50]. From the analysis of the "average" exposure for this residue in a series of proteins (see Materials and Methods section), tryptophan residues generally expose an average surface of 49 ± 36 Å^2^. By analysing for comparison a non-redundant set of interfaces in protein complexes, we found that tryptophan residues have a probability p = 0.175 to be involved in an interface, but those exposing at least 80 Å^2^ have a much higher probability (p = 0.565). Taken together, these observations suggest that this tryptophan could play a role in the complexation of partner proteins. As can be seen in Figure 4A in the p53_human structure, Trp146 is the last residue of S4, which is directly contacting S1. This Trp is conserved in the mouse ortholog and in most mammalian species, and it is mutated to a lysine in the more stable proteins p53_worm (Figure 4B), p63 and p73, as well as to an arginine in the p53_chicken sequence. Another interesting amino acid substitution occurring in the p53_worm protein with respect to the human one, is located on strand S3 where p53_human Asn131 is replaced by a lysine (Lys 251 in the worm sequence), as shown in Figure 4A and 4B. The same substitution is observed in human p63 and human p73. The substitution does not affect directly the interaction between strands S2 and S3, but in the p53_worm structure the lysine is forming a salt bridge with Glu227 on strand S1, thus stabilizing the secondary structure content of the protein and contributing to the global stability of the β-barrel core.

#### Helix insertion in *C. elegans* p53

In the region reported to form the 5’ dimer of dimers interface, the p53_worm presents a five residues insertion of helical content. This helix buries the hydrophobic residues Val293 and Ile297 (27 and 15 Å^2^ SASA, respectively) towards the core of the protein. In addition, the guanidinium group of the neighbouring Arg298 is in contact with the backbone nitrogen atom of His391 on strand S11 (Figure 4C and 4D).

#### Backbone hydrogen bond wrapping

We investigated whether the wrapping of backbone hydrogen bonds could play a role in the experimentally observed difference in stability for these proteins and therefore calculated the average BHB wrapping ρ for each of the p53 family of proteins studied here (Table 3). The p53_worm structure shows the best BHB wrapping amongst the analysed proteins, with 32 hydrophobic carbonaceous groups on average in the desolvation area of each BHB. This observation correlates with the higher thermodynamic stability observed for this protein. Similarly, the p53_fly structure also displays a very good wrapping index with ρ = 31.6. On the other hand, p53_human shows one of the worst wrapping indices along with p53_chicken (ρ = 28.7 and ρ = 28.5, respectively). This trend is in line with the differences in stability between human and invertebrate p53 orthologs. In Table 3 the number of Vbonds (poorly wrapped BHBs) calculated for each structure is also reported. It is worth noticing that the only Vbond common to all 7 proteins involves the helical segment implicated in zinc-ion binding. These data suggest that a better wrapping of backbone hydrogen bonds could be one of the adopted mechanisms to achieve more stability in some of the studied species.

**Table 3:** Backbone hydrogen bond wrapping.

**Protein**	**Average BHB wrapping**	**Number of vulnerable bonds**
p53_human	28.7	9
p63_human	30.0	5
p73_human	29.1	8
p53_mouse	30.6	8
p53_fly	31.6	3
p53_chicken	28.5	7
p53_worm	32.0	4

### Molecular Dynamics Simulations

#### Structural stability in Molecular Dynamics Simulations

**Figure 5:**
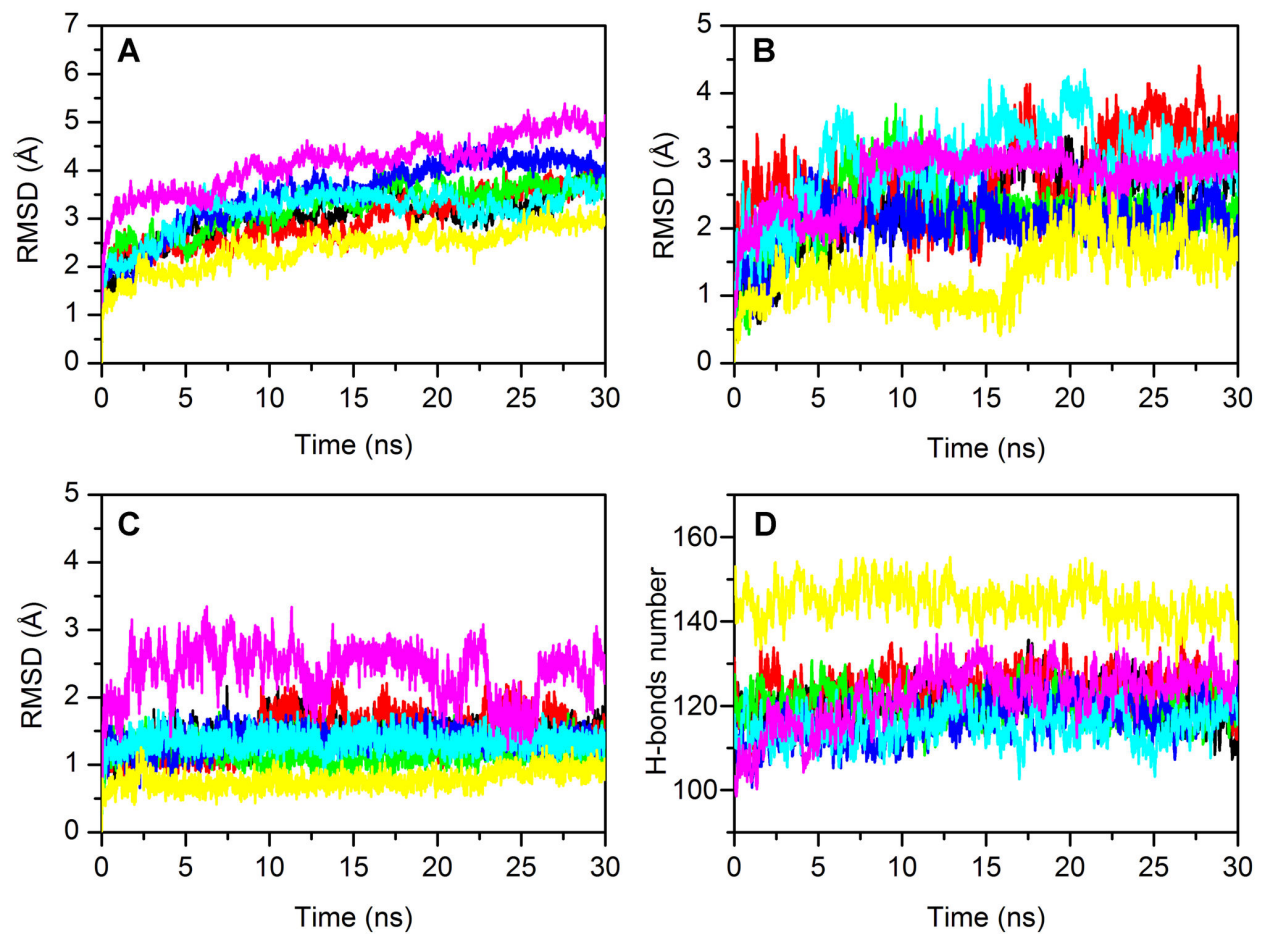
Cα atoms RMSD of the investigated proteins (A), loops 1 (B) and 3 (C) and number of intramolecular H-bonds of the proteins (D). Cα RMSD of (A) the proteins, of (B) L1 loop, (C) L3 loop and (D) number of intramolecular H-bonds of p53_human (black line), p63 (red line) and p73 (green line), p53_mouse (blue line), p53_chicken (cyan line), p53_fly (purple line) and p53_worm (yellow line), computed from the starting structures as a function of the simulation time.

We performed 30 ns of Molecular Dynamics (MD) simulations on all the studied systems. In Figure 5A the overall root mean square displacement (rmsd) of the C-alpha atoms is reported. The p53_worm simulations have the lowest rmsd, while the p53_fly structure undergoes the largest rmsd, but as this is a modelled structure, we expect some fluctuations. All the other structures stabilised at an rmsd of about 3.0 Å. A closer look at the local structure deviations responsible of these values can be found in the analysis of the loop 1 (L1) and loop 3 (L3) (Figure 5B and 5C), both of which are involved in DNA binding [62]. For all the studied proteins L1 seems to be more flexible and shows different motilities within the set. Larger fluctuations for this loop are observed for p53_chicken and p63 human, followed by p53_worm. One of the reasons, at the atomistic detail, for the high stability of p53_worm is the high number of intra-molecular hydrogen bonds, as reported in Figure 5D. This structure shows an average number (calculated on the last ns of trajectory) of 141 vs. only 115 observed for p53_human, the protein with the lowest number of intra-molecular H-bonds. The other proteins show each about 120 H-bonds on average. This trend is reflected in the analysis of the energy components for all the simulated species (Table 4). The p53_worm shows the most favourable Coulomb energy term over the last 2 ns. This term reflects the hydrogen bond contribution to the internal energy of the molecule. The other species show a very similar behaviour for this term, except for the p53_worm protein that has a marginally more favourable Coulomb intra-molecular interaction. p53_human shows the lowest value for the 1-4 Lennard-Jones contribution, with p53_worm and p53_fly showing favourable contributions.

**Table 4:** Energy decomposition terms.

**Protein**	**Energy term**	**0-2 ns kJ/mol**	**28-30 ns kJ/mol**	**ΔE kJ/mol**
p53_human	Coulomb	-8162 (144)	-8353 (163)	-191
	LJ-SR	-4837 (69)	-4939 (65)	-102
	LJ-LR	-223 (2)	-227 (2)	-4
	LJ-14	-163 (40)	-190 (40)	-27
p63_human	Coulomb	-8420 (229)	-8636 (140)	-216
	LJ-SR	-4909 (68)	-5031 (65)	-122
	LJ-LR	-229 (2)	-236 (2)	-7
	LJ-14	-182 (41)	-209 (39)	-27
p73_human	Coulomb	-8264 (166)	-8341 (133)	-77
	LJ-SR	-4766 (68)	-4754 (67)	12
	LJ-LR	-220 (2)	-222 (2)	-2
	LJ-14	-160 (40)	-203 (38)	-43
p53_mouse	Coulomb	-7807 (184)	-8257 (141)	-450
	LJ-SR	-4651 (67)	-4754 (66)	-103
	LJ-LR	-213 (2)	-217 (2)	-4
	LJ-14	-151 (38)	-204 (37)	-53
p53_chicken	Coulomb	-8022 (182)	-8308 (134)	-286
	LJ-SR	-4750 (63)	-4779 (65)	-29
	LJ-LR	-218 (2)	-216 (2)	2
	LJ-14	-164 (39)	-204 (39)	-40
p53_fly	Coulomb	-8114 (157)	-8642 (146)	-528
	LJ-SR	-4909 (73)	-5099 (67)	-190
	LJ-LR	-227 (2)	-239 (2)	-12
	LJ-14	-229 (40)	-261 (39)	-32
p53_worm	Coulomb	-9359 (171)	-9476 (168)	-117
	LJ-SR	-5374 (72)	-5305 (70)	69
	LJ-LR	-245 (2)	-249 (2)	-4
	LJ-14	-196 (39)	-275 (39)	-79

**Figure 6:**
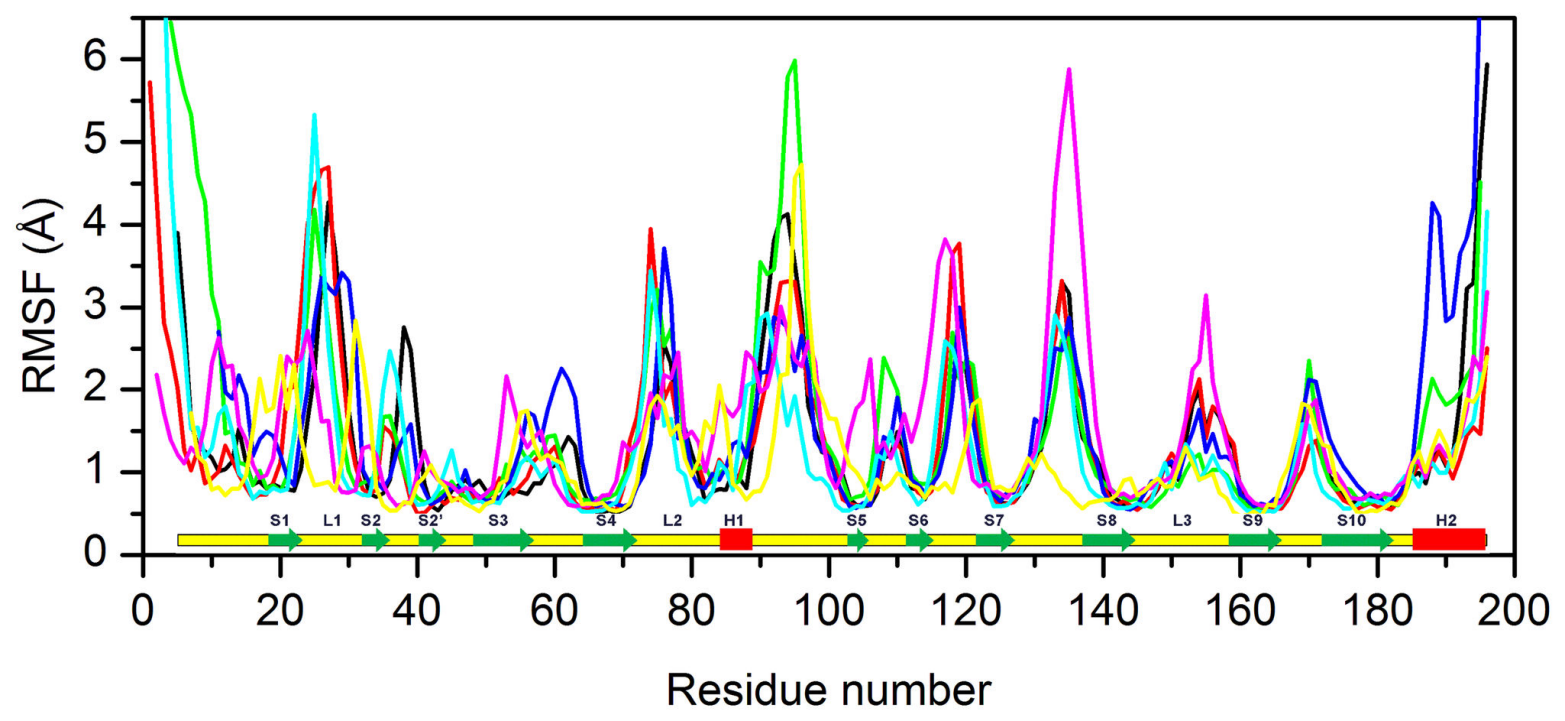
RMSF of Cα atoms of the proteins. RMSF of Cα atoms of p53_human (black line), p63 (red line) and p73 (green line), p53_mouse (blue line), p53_chicken (cyan line), p53_fly (purple line) and p53_worm (yellow line). The residue number refers to p53_human. Secondary structure of p53_human is displayed along the sequence (bottom panel): α-helices and β-strands are shown by red rectangles and green arrows, respectively.

In Figure 6 the root mean square fluctuation (rmsf) of the C-alpha atoms of all the studied proteins is reported. The secondary structure highlighted at the bottom refers to the p53_human structure. Major fluctuations are observed for the regions L1-S2-S3, H1-S6 and S7-S8. In particular p53_human, p63, p73 and p53_worm show high fluctuations in proximity of H1, suggesting a conformational plasticity in the DNA binding region. In particular the p53_fly structure displays high rmsf value in the segment linking S7 and S8.

By looking at the 'sausage' plot figures (Figure S2) calculated over the simulated structures, one can observe the mobility being concentrated on the three loops with p53_worm showing a less mobile L2, with more helical secondary structure as observed before [26].

#### Stability of secondary structure elements

The α-helix and β-sheet contents in the MD simulations are presented in Table 1. Compared to the starting structures, p53_human loses the largest fraction of α-helix content compared to the other species. All the proteins optimise their β-sheet content during the simulation, with the systems p73, p53_chicken and p53_fly showing the most dramatic changes. The β-sheet content is mainly attributable to the S1 strand (residues 110-112 in p53_human). S1 is considerably elongated in the p53_worm protein and forms 6 H-bonds with the backbone of strand S4. These are maintained during the simulated time. On the other hand, in the p53_human protein, the short strand S1 forms only 4 H-bonds with S4.

Comparing the MD averaged values with the experimental CD ones (Table 1 values at 20 °C) we observed a striking agreement for the α-helix and β-sheet contents for the p53_human, p63_human and p53_worm species (values in bold). Even more remarkable is that the MD values for these species are very close also to the ones obtained at the end of the heath/cool cycle. In fact for all the other species, the secondary structure contents are closer to the finally re-cooled experimental values. These results are supporting the stability of these domains as measured by MD simulations. We investigated the molecular details playing a role in this.

**Figure 7:**
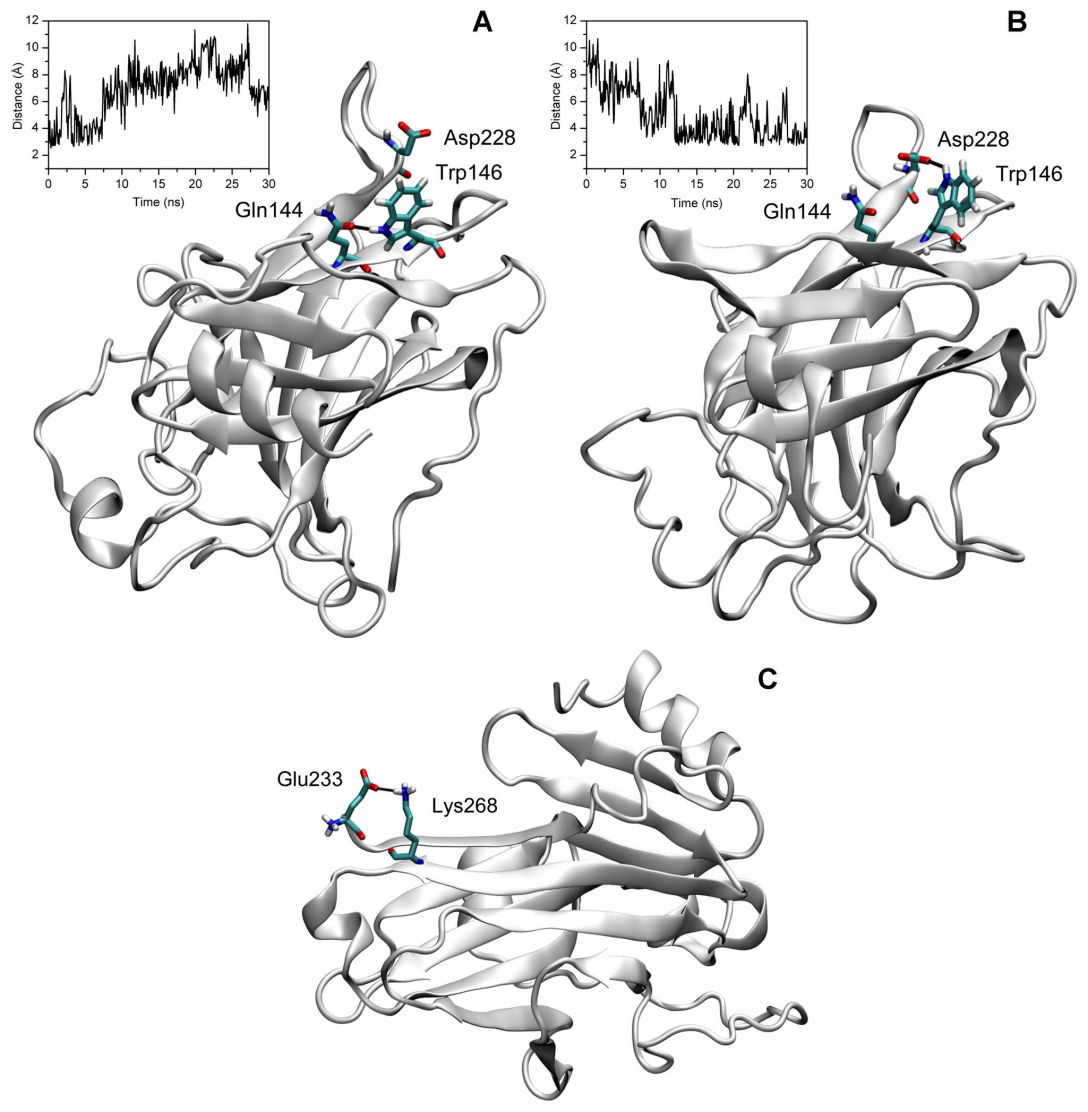
Cartoon representation of p53_human (A, B) and of p53_worm structures (C). Cartoon representation of (A) starting and (B) average final structures of p53_human, highlighting the formation of H-bonds involving Gln144, Trp146 and Asp228 residues (shown as licorice). The insets show the time evolution, over the entire simulation time, of the distance between the hydrogen bond-forming atoms: (A) Gln144-Trp146 and (B) Trp146-Asp228. (C) Cartoon representation of p53_worm structure, showing the formation of a salt bridge between Glu223 and Lys268 residues (shown as licorice).

The overall stability of the β-barrel of the p53_worm core is dependent on the presence of salt-bridges present on one side of the β-sheeted surface. These are between the residues Glu227 and Asp229. As mentioned before, the presence of Lys266 in p53 worm instead of a tryptophan (Trp 146 in p53_human), results in an increased stability of the DBD domain. In the starting structure of p53_human, Trp146 is hydrogen bonded with Gln144 of the same strand (S4), but this bond is lost after about 7-8 ns of simulation (Figure 7A). After about 12 ns the Trp 146 residue interacts with Asp228, which is located at the beginning of S8 (Figure 7B). During the simulation a salt-bridge network is formed involving the strands S1-S3-S4 due to interactions between the residues Glu227, Asp229, Lys251 and Lys266. This dynamically changing network of interactions persists for periods of the trajectory and involving these residues in couples or sometimes as a group of three (Figure S3). Interestingly, a stable salt bridge is formed between residues Glu223 and Lys268 that seems to act as a clip between the S1 and S4 strands and 'zips' the complementary strands together (Figure 7C). The most striking fact is that no salt bridge is present in the p53_human structure, in fact no charged residue is present within the first 52 residues of this domain, which include strands S1-S2-S3-S4. The p53_human surface is therefore more ‘fragile’ and less stabilised by intra-molecular interactions.

#### Helix insertion in p53_worm

**Figure 8:**
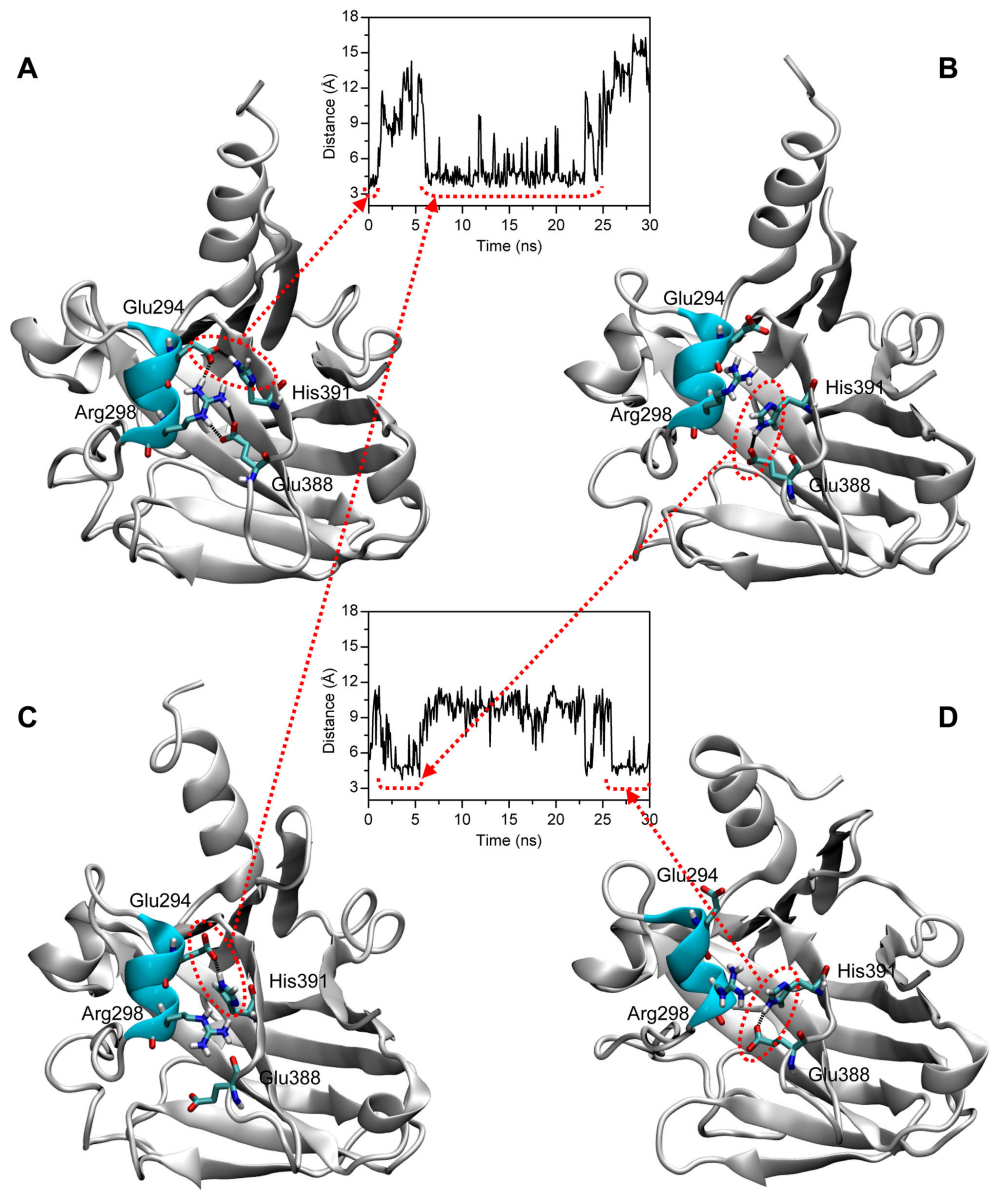
Interactions of the residues of helix insertion in p53_worm. In the starting structure (A), Arg298 and His391 are in close contact, probably forming a cation-π interaction that is lost during the simulation. Glu294 is in close contact with His391 (A), forming a salt bridge that persists for quite long time (C), but that for short time is swapped for a salt bridge with Glu388 (B, D).

As previously mentioned, in the area corresponding to the 5’ dimer of dimers interface (blue shaded area, Figure 3) the p53_worm protein shows an insertion of 8 residues, 5 of them forming a α-helix. The rmsd of this helix is quite small (Figure S4), suggesting a stable arrangement in the 3D structure. At the beginning of the simulation, Arg298 and His391 were in close contact but this is lost during the simulated time. A residue of the inserted helix, Glu294, forms a salt bridge with His39 that persists for a period of the simulated time (Figure 8A and 8C) and then forms a hydrogen bond interaction with Glu388 (Figure 8B and 8D).

#### DNA binding region electrostatic features of the isolated proteins

**Figure 9:**
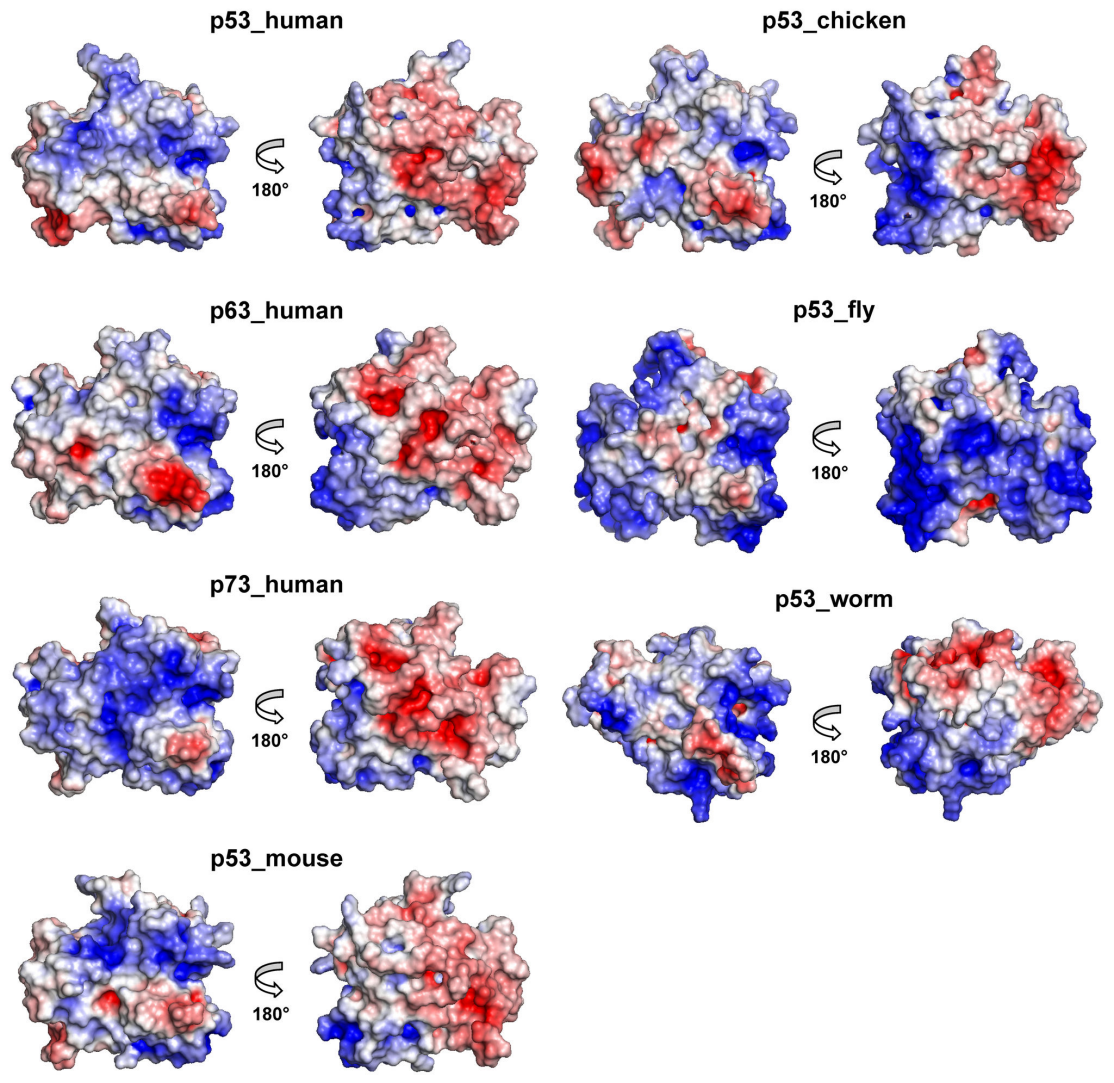
Electrostatic surfaces and charge distribution of the proteins. Comparison of the electrostatic surface of the starting structures of the proteins, two views are shown for each protein.

A map of the electrostatic surface of the p53 family of proteins is reported in Figure 9, where two views are shown for each protein. Generally, the most conserved DNA-binding region is more positively charged (blue), while the least conserved surface is generally more negatively charged on all the proteins (red), except for p53_worm.

The principal component analysis [63] of the simulated trajectories shows that between 40 and 60% of the motion is due to the first eigenvector, for all the studied proteins (Figure S5). By showing the mobility by the 'sausage' representation we can visualise the overall displacement observed in the simulation on the structure topology (Figure S2). It can be noted that for almost all proteins the surface facing DNA is the most mobile with variability in the flexibility, despite this being the most conserved region amongst the studied proteins.

## Discussion

It is well known that the p53 human protein is particularly unstable when compared to homologs within the same evolutionary family; similarly it has been reported that stability and aggregation have at least as great a role in protein evolution as in cellular and organismal function [10].

In the present study, we set out to investigate the molecular basis of thermal stability of the p53 protein family. We selected representatives of p53 invertebrates as well as p53 vertebrate species. In addition we analysed the p53 homologous proteins p63 and p73. We used CD analyses coupled with structure-based homology modelling and Molecular Dynamics simulations.

A recent investigation reported on the, simulated p53 cancer mutation spectrum using dipeptide composition across the p53 protein family [64]. The authors found that the evolution of dipeptide composition in the p53 is reversed by the so-called “hot spot mutations”. The gain-of function mutants are suggested to relate to p53 ancestral function, which was lost during evolution. Here we do not focus on cancer-related mutations, but only on the structural stability of different p53-related species, and on the effect of naturally occurring substitutions amongst these on the structural stability of the DNA–binding domain.

We observed high melting temperatures for p53_worm, closer to the ones exhibited by p73 human and p63 human, and in a different range than p53_human proteins respectively. We offer a rationale for the observed high stability of the species studied based on structural and bioinformatics analyses of the different DNA binding domains.

The thermal stability of the proteins studied here by CD and reported as apparent melting temperatures, Tm, is in agreement with the stabilities of similar constructs of these proteins studied by differential scanning calorimetry [23]. The trend is that p53 proteins from invertebrates p53_worm (47.8 °C) and p53_fly (47.3 °C) have higher stability than the p53 from vertebrates such as p53_human (41.5 °C) and the p53_mouse, (44 °C) with the exception of the p53_chicken (49 °C). Mammalian p53 proteins represented in this work by p53_human (41.5 °C) and p53 mouse (44.0 °C) have lower apparent Tm than the p53 paralogs p63 human (59 °C) and p73 human (49.5 °C). We found, as others, that the stabilities of the p53 proteins from p53_worm and p53_fly are closer to human p63 and human p73 than to p53_human [23]. These data suggest that p53_human has lower stability relative to the evolutionary close homologs p63 and p73 and that this is probably intrinsic to its specific oncogenic function and resulting into wide spread cancer-related mutations mapped to the DNA-binding domain investigated here. Kinetic stability studies of p53 proteins revealed that homoeothermic species such as p53_human, p53_chicken and p53_mouse, have apparent melting temperatures that correlate with the body temperatures of these species and that the proteins are kinetically unstable as shown by the half-life values ranging from 10-20 minutes at the body temperatures of these species, which are 40-44, 36-37, and 36-37 °C, respectively [23].

The analysis of the conformational stability of the studied proteins through structural bioinformatics analyses and through Molecular Dynamics simulations has highlighted some interesting structural features for some of the ortholog species.

The observed structural stability of the p53 fly DBD (lowest rmsd), had been reported by others in a comparison with only the p53_human protein [26]. Here we offer further insight into this structural stability, which emerges from the comparison with the other species. Stabilising mutations in loop L1 have been shown to have a direct effect on the thermal stability of the p53 proteins [26], therefore this is a crucial secondary structure element in the design of more stable p53 proteins. According to our simulations one of the reasons, at the atomic level, of the intrinsic stability of p53 worm is to be found in the number of intra-molecular hydrogen bonds, as we reported in Figure 5D.

A high number of intra-molecular hydrogen bonds are also found in the most thermally stable protein, p63, for the other species thermal stabilities trend it is very difficult to extract a clear determinant from the simulations.

We want to highlight the importance of the insertion of 8 residues in the p53_worm protein forming a α-helix stretch, very stable during the simulated trajectories. We observed some minor rearrangements of residues belonging to this inserted helix, and new intra-molecular contacts (Glu294-His391) formed during the simulation, contributing to a further structural stability of the molecule. These contacts contribute to keep the position of the inserted helix, but also in burying at the same time the putative dimer interface. This is in line with previous observations reporting that DNA-binding mode of p53_human and its *C. elegans* ortholog showed significant differences, despite their conserved biding specificity [32].

We have compared the secondary structure content calculated over the production phase of the Molecular Dynamics simulations with the values from the CD spectra analyses (Table 1). Interestingly, our results are remarkably close to the experimental values for the most stable specie *C. elegans* and for the least stable *Homo sapiens*. For the other species we do not observe the increase in the α-helix content as found in the experiments at 20 °C.

From the structural alignment of the species considered here, it appears that regions of the isolated DBDs and responsible for dimerization and tetramerization are less conserved, and particularly so for the evolutionary more distant species and reported as more stable, p53 worm and fly proteins [65]. Interestingly, for these proteins the dimeric organizations have been shown to be more likely to dissociate into monomers [66]. This raises the issue whether the core domain of these p53 proteins would tetramerize or dimerize at all in absence of the C-terminal oligomerization domain. It was reported based on a DNA-free p53 model that Arg174 is important for dimerization, while the corresponding amino acid residue Lys174 in the hypoxia-tolerant subterranean mole blind rat *Spalax* prevents such interactions. Notably, similar mutations observed in human tumours favour growth arrest instead of cell cycle death [16].

We also analysed the backbone hydrogen bond (BHB) wrapping content in the structures analysed here. This contribution has been shown to correlate with the stability of proteins in a number of previous structural analysis [44,67]. These values indicate that the most stable protein in our analyses, the p53_worm protein, shows the better BHB wrapping values, followed by p53_fly, while p53_human shows the lowest value for this contribution. These results correlate with the relative thermodynamic stability of these proteins and again support the hypotheses that the proteins more stabilised intra-molecularly are the least prone to oligomerise. From the analysis of the overall displacement experienced in the simulations (Figure S2), we observed the surface facing DNA as the most mobile in spite of being the most conserved region amongst the studied proteins. Therefore there must be a balance between the intra-molecular stability and the flexibility exerted in the DNA binding region playing a role in the protein-protein and protein-DNA binding properties of these molecules and hence in their biological functions. These findings are all the more relevant in the light of current efforts to identify cellular networks and interactomes on a genome-wide scale [68].

In fact, in a recent genome-scale protein interaction profile studies of the p53_worm protein, 91 new interactions were identified [8]. Further binding studies of mammalian orthologs of p53_worm protein interactors to p53, p63 and p73 identified that 90% were able to bind to one or more p53 protein family member implying that these interactions are evolutionarily conserved. These findings are particularly exciting as they may shed further light on the p53 family of proteins and their role in the cellular signalling networks. We observe sequence deletions in the DNA binding domain of p53_worm and some hydrogen bonding properties typical for this ortholog that could be playing a role in the exerted binding properties.

By studying the multiple structure alignments of the analysed species, we highlight here the presence of a lysine residue in the p53_worm sequence (Lys266), conserved also in *Homo sapiens* p63 and p73, instead of a tryptophan (Trp146) in p53_human. This residue supported the β-sheet elongation observed in the p53_worm DBD domain. Importantly, Trp146 is unusually exposed to the solvent in the p53_human structure. The latter has in fact been used as probe for fluorescent measurements in protein denaturation studies [65]. We hypothesize here that this residue could have a crucial role in protein dimerization and protein-protein interactions and therefore be important in the functional activity of p53_human. Our results could be exploited in the design of p53 mutants with enhanced stability and/or of proteins with specific oligomerization properties.

## Supporting Information

Figure S1 **Superposition of the model and X-ray structures of p63 and p73 proteins.** (A) Superposition of the model structure of p63 (cyan) with the X-ray structure (purple) pdb code 3US0. (B) Superposition of the model structure of p73 (gray) with the X-ray structures having pdb codes 3VD2 (blue), 2XWC (red), 4A63 (orange) and 4G82 (green).
(TIFF)

Figure S2 **Sausage plot of the proteins.** Sausage plot indicating the extent of protein chain motion along the first eigenvector during the MD simulations of p53_human (A), p63 (B), p73 (C), p53_mouse (D), p53_chicken (E), p53_fly (F) and p53_worm (G). Coils are coloured in yellow, β-sheets are coloured in green and α-helices are coloured in red.
(TIFF)

Figure S3 **Salt-bridge network in p53_worm.** During the MD simulation a salt-bridge network is formed involving the residues of the strands S1-S3-S4 of p53_worm. This is a dynamically changing network of interactions between the side chains of the residues Glu227, Asp229, Lys251 and Lys266, involving these residues sometimes in group of three (A, B) or sometimes in couples (C, D).
(TIFF)

Figure S4 **Cα atoms RMSD of helix insertion in p53_worm.** RMSD of the Cα atoms of helix insertion in p53_worm computed from the starting structure as a function of the simulation time. The RMSD is quite small, suggesting a stable arrangement of the structure.
(TIFF)

Figure S5 **Percentage of eigenvector contributions.** Percentage of contribution of the first 10 eigenvectors to the motion of the proteins during the simulated trajectories of p53_human (A), p63 (B), p73 (C), p53_mouse (D), p53_chicken (E), p53_fly (F) and p53_worm (G).
(TIFF)
