# Supplementary figures and images for: Structure and Stability Insights into Tumour Suppressor p53 Evolutionary Related Proteins
Source: PLoS One. 2013 Oct 4;8(10):e76014. doi: 10.1371/journal.pone.0076014 (PMC3790848; doi:10.1371/journal.pone.0076014)

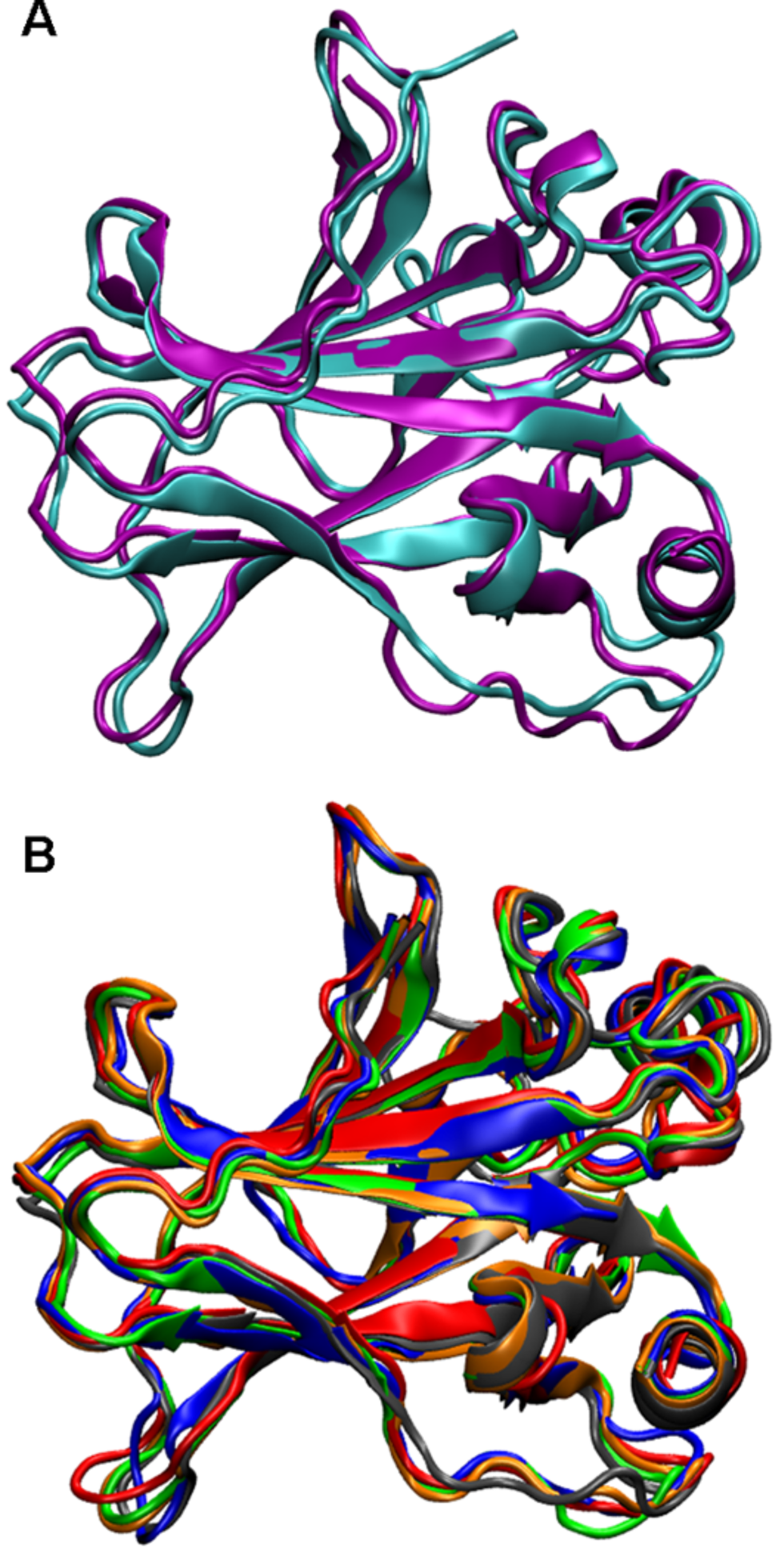

Supplement: Figure S1 — Superposition of the model and X-ray structures of p63 and p73 proteins. (A) Superposition of the model structure of p63 (cyan) with the X-ray structure (purple) pdb code 3US0. (B) Superposition of the model structure of p73 (gray) with the X-ray structures having pdb codes 3VD2 (blue), 2XWC (red), 4A63 (orange) and 4G82 (green). (TIFF) [file pone.0076014.s001.tiff]

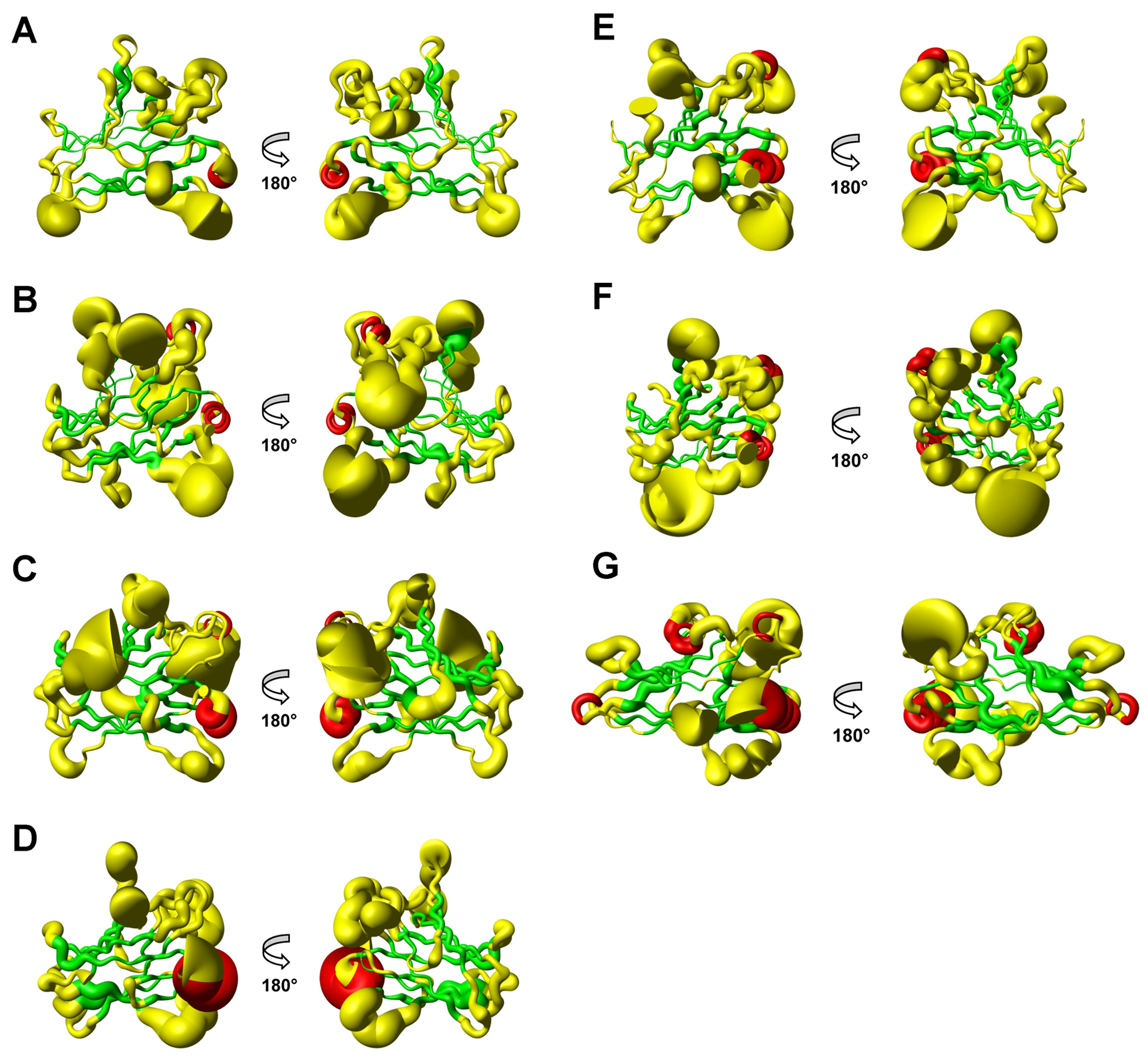

Supplement: Figure S2 — Sausage plot of the proteins. Sausage plot indicating the extent of protein chain motion along the first eigenvector during the MD simulations of p53_human (A), p63 (B), p73 (C), p53_mouse (D), p53_chicken (E), p53_fly (F) and p53_worm (G). Coils are coloured in yellow, β-sheets are coloured in green and α-helices are coloured in red. (TIFF) [file pone.0076014.s002.tiff]

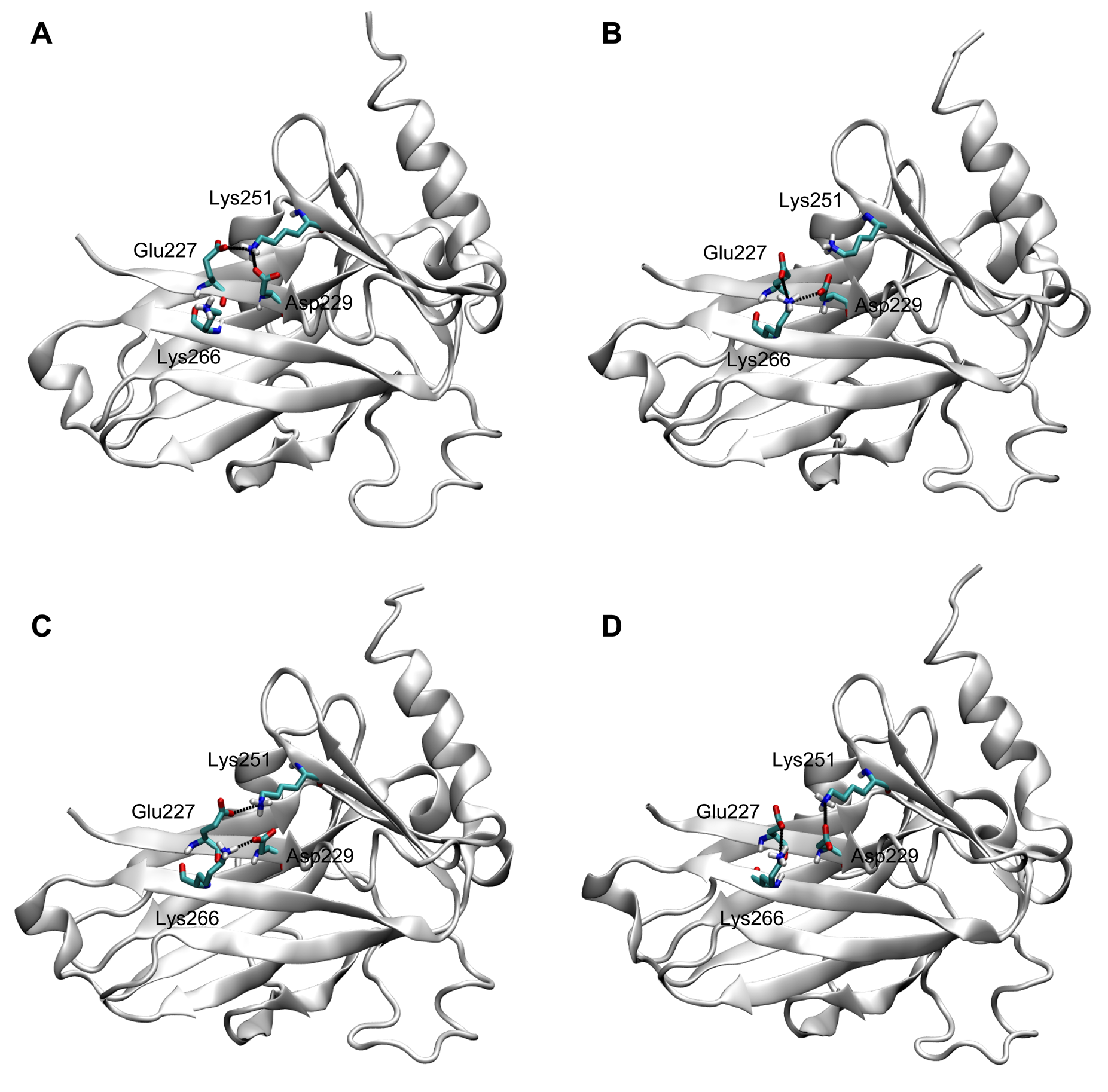

Supplement: Figure S3 — Salt-bridge network in p53_worm. During the MD simulation a salt-bridge network is formed involving the residues of the strands S1-S3-S4 of p53_worm. This is a dynamically changing network of interactions between the side chains of the residues Glu227, Asp229, Lys251 and Lys266, involving these residues sometimes in group of three (A, B) or sometimes in couples (C, D). (TIFF) [file pone.0076014.s003.tiff]

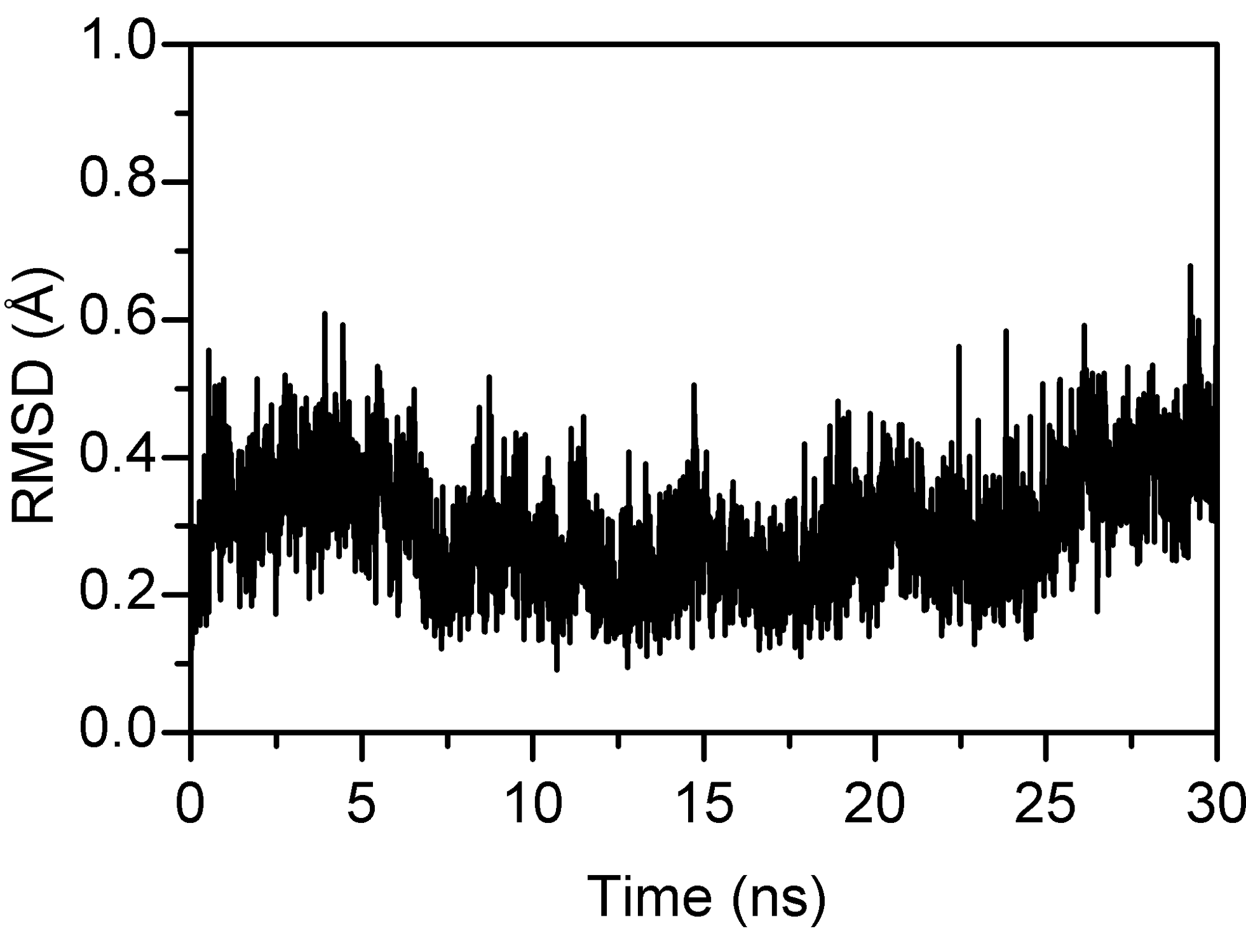

Supplement: Figure S4 — Cα atoms RMSD of helix insertion in p53_worm. RMSD of the Cα atoms of helix insertion in p53_worm computed from the starting structure as a function of the simulation time. The RMSD is quite small, suggesting a stable arrangement of the structure. (TIFF) [file pone.0076014.s004.tiff]

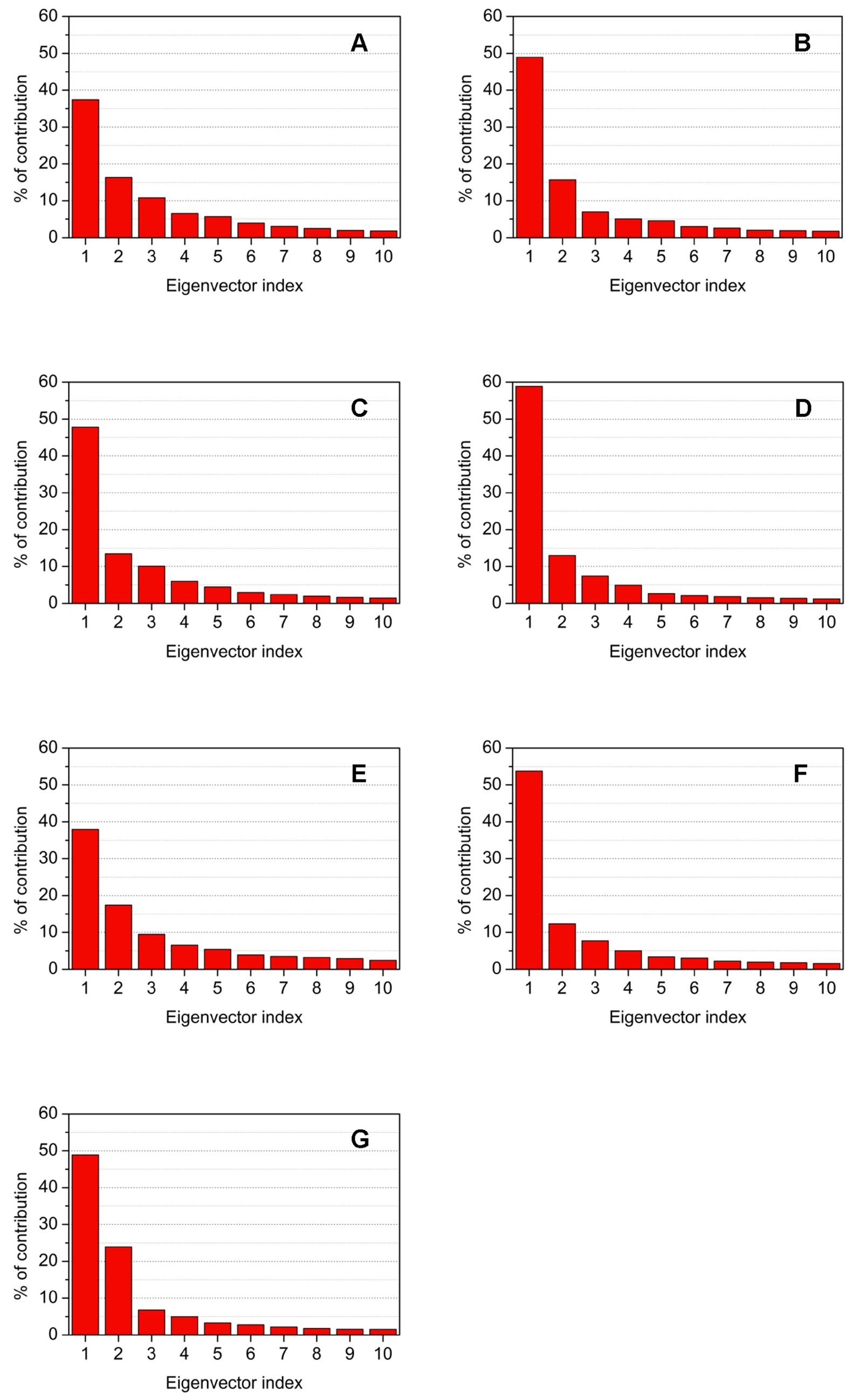

Supplement: Figure S5 — Percentage of eigenvector contributions. Percentage of contribution of the first 10 eigenvectors to the motion of the proteins during the simulated trajectories of p53_human (A), p63 (B), p73 (C), p53_mouse (D), p53_chicken (E), p53_fly (F) and p53_worm (G). (TIFF) [file pone.0076014.s005.tiff]
